# Potential of *Streptomyces rochei* G-6 for Biocontrol of Cucumber Wilt Disease and Growth Enhancement

**DOI:** 10.3390/jof10120885

**Published:** 2024-12-20

**Authors:** Chengyu Zhu, Xin Li, Yan Gao, Xueying Yang, Yuliang Gao, Kuihua Li

**Affiliations:** 1 Agricultural College, Yanbian University, Yanji 133002, China; 18343699121@163.com (C.Z.); 13043365088@163.com (X.L.); a1695881208@163.com (Y.G.); 17643301229@163.com (X.Y.); 2 Vegetable Research Institute of Yanbian Agricultural Sciences Academy, Longjing 133400, China

**Keywords:** cucumber wilt disease, *Streptomyces rochei*, biocontrol agent, cucumber grow promotion

## Abstract

Cucumber wilt disease, caused by *Fusarium oxysporum* f. sp. *cucumerinum* (FOC), is a major threat to cucumber production, especially in greenhouses. This study used a fermentation product derived from a new strain of *Streptomyces rochei* (G-6) to investigate the potential for biocontrol of cucumber wilt disease and the effect on promoting cucumber growth. In the first experiment, the inhibitory effect of *S. rochei* G-6 fermentation product (SGFP) on FOC growth was evaluated, then the effect of SGFP on wilt incidence and severity, as well as cucumber growth, antioxidant system, and soil nutrient conversion capacity were investigated. The results showed that SGFP inhibited FOC growth by 85.3% in the antimicrobial experiment. In the potting experiment, the incidence rate in the FOC group reached 88.7%, but it was only 56.0% in the SGFP1 group and 64.7% in the SGFP2 group, indicating the efficient inhibitory effect of SGFP on cucumber wilt, with the biocontrol effect of SGFP1 being higher than that of SGFP2. In addition, the disease index decreased significantly (*p* < 0.05) in both SGFP treatments, which was significantly (*p* < 0.05) lower in the SGFP1 group than in the SGFP2 group, indicating that pre-treatment was better than post-treatment in reducing the disease severity. In addition, SGFP promoted the growth of cucumber seedlings, as indicated by indicators related to the growth of aboveground and underground parts. Furthermore, the activities of antioxidant enzymes (superoxide dismutase, peroxidase, and catalase) in the cucumber seedlings increased after SGFP treatment and the malondialdehyde level was decreased, indicating a reduction in oxidative stress. SGFP also improved the soil nutrient conversion capacity by increasing the activities of urease, phosphatase, and sucrase, which may enhance nutrient uptake by cucumber seedling. The findings of this study suggest that SGFP is an effective biocontrol agent against cucumber wilt and also promotes cucumber growth by regulating the antioxidant system and soil environment, and its application is a promising solution to reduce wilt incidence in cucumber production.

## 1. Introduction

Cucumber wilt is a soil-borne disease caused by the fungus *Fusarium oxysporum* f. sp. *cucumerinum* (FOC). It can occur throughout the growing period, with peak incidence during the flowering and fruiting stages [1]. Typical symptoms are wilting and, as the disease progresses, whole plants may collapse [2]. In China, the incidence of cucumber wilt has been increasing year on year, typically ranging from 10–30%, and in severe cases over 50%, resulting in a 10–50% reduction in cucumber yield, and in extreme cases, complete crop loss, leading to significant economic losses [3].

Currently, strategies such as selection of disease-resistant cultivars, agronomic practices, chemical treatments, and biocontrol methods are used to control wilt disease in cucumber production [4]. Since the 1980s, the biocontrol approach has gradually gained prominence, with its primary mechanism focusing on inhibiting pathogenic growth using antagonistic microorganisms or their metabolites [5]. Biocontrol can minimize the development of drug resistance in pathogens and can also provide the advantages of environmental sustainability, long-term efficacy, and the maintenance of plant product quality [6]. Various microbial agents, such as bacteria, fungi, and actinomycetes, have biocontrol effects on plant pathogens, but the efficiency differs among them. Therefore, screening for efficient antagonistic microbial resources has become a major research focus in this area [7,8].

The genus *Streptomyces*, a group of actinomycetes, has been shown to be antagonistic to various plant diseases [9,10,11]. *Streptomyces* sp. WP-1 inhibits many pathogenic fungi such as *Rhizoctonia solani*, *Candida albicans*, *Phytophthora infestans*, and *F. oxysporum*, exhibiting particularly strong biocontrol effects on wilt disease caused by *F. oxysporum* [12]. Notably, *Streptomyces* can produce abundant secondary metabolites, including antibiotics, antimicrobials, and other bioactive substances [13,14], which play a crucial role in inhibiting pathogens responsible for wilt disease. For instance, the fermentation broth of *Streptomyces* sp. HAAG3-15 [15] and *Streptomyces* sp. JCK-6131 [16] exerted great inhibitory effect against cucumber wilt disease, along with plant growth-promoting role. However, the antimicrobial activities of *Streptomyces* vary depending on the species and strains [17]. Kaur et al. [17] found that *Streptomyces* sp. MR14 could inhibit 13 different fungal pathogens, but its inhibitory activity against *F. oxysporum* was the lowest among those tested, suggesting that *Streptomyces* used for wilt biocontrol should be carefully selected. To date, scientists continue to search for new biocontrol agents to combat pathogen resistance, improve control efficacy, and promote sustainable agriculture.

*Streptomyces rochei* typically exhibits filamentous growth and can form characteristic spores commonly found in soil [18]. This species is capable of producing a variety of compounds such as antimicrobials, auxins, cytokinins, phenylacetic acid, and succinic acid [19,20]. Unfortunately, the biocontrol effect of *S. rochei* has not been extensively studied, with only a few reports available on the subject. For example, Kanini et al. [21] found that *S. rochei* ACTA1551 possessed inhibitory activity against *F. oxysporum* f. sp. *lycopersici* and showed an enhancement effect on plant growth of tomato. Manhas et al. [22] showed that *Streptomyces* sp. M4, a sister species of *S. rochei*, can produce salvianolic acid B, which efficiently inhibited various fungal pathogens, including wilt-causing *Fusarium* spp. These findings demonstrate that *S. rochei* may be a promising antimicrobial candidate, and are valuable for agricultural applications [23].

Our team previously isolated a new strain of *S. rochei*, G-6, from FOC-infected cucumber soil [24] and found that cucumber wilt could be inhibited by *S. rochei* G-6 fermentation product (SGFP). This finding implied the potential biocontrol effects of SGFP on cucumber wilt disease. Therefore, based on the preliminary experiment, this study investigated the inhibitory effect of SGFP on FOC growth in the first experiment. Subsequently, SGFP was used to treat pot-grown cucumber seedlings to explore its biocontrol effects, along with its ability to promote cucumber growth. The aim of this study was to identify a new agent for biocontrol of cucumber wilt and to provide a promising candidate for the development of new pesticides.

## 2. Materials and Methods

### 2.1. Experimental Microorganisms and Their Maintenance, and SGFP Preparation

FOC was provided by researcher Rong-Jun Guo of the Chinese Academy of Agricultural Sciences. *Streptomyces rochei* G-6, isolated from FOC-infected cucumber soil in Yanji, Jilin Province, China, was identified through biological characterization and 16S rDNA gene sequence analysis by Li et al. [24] and has been deposited at the China Microbial Strain Conservation Center, under CGMCC24046.

Agar plugs (*ϕ* = 5 mm) were obtained from FOC colonies cultured on potato dextrose agar (PDA) medium (200 g/L potato + 20 g/L glucose + 20 g/L agar) at 28 °C according to the method of Dong et al. [25] and used in the antimicrobial experiment. For preparation of FOC suspension for using in pot experiment, the agar plugs were inoculated into the potato dextrose broth and suspension cultured at 180 rpm at 28 °C for five days to obtain a suspension of 1 × 10^6^ CFU/mL FOC.

To obtain SGFP, *S. rochei* G-6 was cultured in a medium composed of 10 g/L glucose, 3 g/L peptone, 2.5 g/L sodium chloride, 2 g/L calcium carbonate, and 10 g/L wheat, and incubated at 28 °C for seven days under shaking at 180 rpm. The resulting suspension (1 × 10^7^ CFU/mL) was then centrifuged at 4500 rpm, and the supernatant (i.e., SGFP) was collected. To verify the presence of *S. rochei* G-6 in SGFP, a spread plate method was then conducted according to Chen et al. [26]. After 24 h of incubation at 37 °C, no bacterial growth was observed on the plates spread with SGFP. Therefore, SGFP was used for potting experiments.

### 2.2. Inhibitory Effect of SGFP on FOC

In this experiment, the method of Katsumata et al. [27] was used. Briefly, a Petri dish filled with 20 mL of PDA medium was prepared, and agar plugs of FOC (*ϕ* = 5 mm) was inoculated in the center of the medium. Subsequently, two pieces of filter paper (L × W = 3 cm × 0.8 cm) soaked in 100 μL of SGFP (the sterilized water as control) were placed on the medium at a distance of 2.5 cm from the agar plugs. After six days of incubation, the FOC area was measured using ImageJ v1.8.0.

### 2.3. Biocontrol Effect of SGFP Against Cucumber Wilt Disease and Its Growth Enhancement

#### 2.3.1. Pot Cultivation and Experiment Design

The experiment was conducted in June 2023 in a greenhouse at Yanbian University, Yanji, China (42.912091° N, 129.48838° E). Pots (*L* × *W* × *H* = 18 cm × 18 cm × 20 cm) were filled with a commercial nutrient substrate (pH, 6.87) (Stanley Agricultural Group Co., Ltd., Linyi, China) containing 15.68 mg/kg organic matter content, 0.34 g/kg total nitrogen (with 64.72 mg/kg available nitrogen), and 0.95 g/kg total phosphorus (with 98.86 mg/kg available phosphorus). The moisture content of the substrate was maintained at approximately 60% during the entire cultivation process.

Seedlings of cucumber ‘Zhongnong 6’, approximately 3 cm long with two expanded leaves each, were prepared. One seedling was planted in each pot. All pots were maintained in a greenhouse at a temperature range of 20–28 °C, with regular watering to keep the substrate moist throughout the cultivation period.

The experiment was designed with four groups, in which the doses of FOC suspension or SGFP and the treatment time were selected based on pro-testing. The detailed groups were as follows: (1) Cont: A control group. After planting the seedlings in pots, 50 mL of water was added separately to the substrate around the root system at the beginning, day 3, and day 6 of cultivation. (2) FOC: A disease group. Before planting, the epidermis of the seedling rhizome was scratched. The seedling was then planted in the pot, and 50 mL of FOC suspension was added to the substrate around the root system to induce wilt disease. After three and six days of cultivation, 50 mL of water was added separately to the substrate. (3) SGFP1: A disease preventive group. The seedling was planted in the pot and 50 mL of SGFP was added to the substrate around the root system. After three days of cultivation, the epidermis of the seedling rhizome was scratched and 50 mL of FOC suspension was added. After three days of FOC inoculation, 50 mL of water was added to the substrate. (4) SGFP2: A disease treatment group. After scratching the epidermis of the seedling rhizome, the seedling was planted in the pot, and 50 mL of FOC suspension was added to the substrate around the root system. After three and six days of cultivation, 50 mL of SGFP was added separately.

After 14 days of cultivation, the incidence of disease status, seedling growth, as well as the antioxidant enzyme activities and malondialdehyde (MDA) levels in seedlings, and nutrient conversion enzyme activities in substrate were determined for each group.

#### 2.3.2. Determination of Disease Incidence

The incidence rate, disease index, and biocontrol efficacy were evaluated based on the following cucumber wilt grading criteria. The following formulas were used to calculate the incidence rate, disease index, and biocontrol efficacy:

Disease grading criteria: Grade 0: Healthy with no symptoms observed; Grade 1: Yellowing or wilting of true leaves and cotyledons does not exceed 50% of the total area; Grade 2: Yellowing or wilting of true leaves and cotyledons exceeds 50% of the total area; Grade 3: Leaves are wilted or dead, with only the growing points surviving; Grade 4: The entire plant is severely wilted or dead.

$$Incidence\;rate\;(\%)=\frac{Incidence\;plant\;number}{total\;plants\;number}\times 100$$

$$\mathrm{Disease}\;\mathrm{index}=\frac{\sum \left(Number\;of\;disease\;greades\times grade\;number\right)}{\left(total\;plant\;number\times the\;highest\;gradenumber\right)}\times 100$$

$$\mathrm{Biocontrol}\;\mathrm{efficacy}\;(\%)=\frac{\left(Disease\;index\;in\;the\;control\;group-disease\;index\;in\;the\;treated\;group\right)}{disease\;index\;in\;the\;control\;group}\times 100$$

#### 2.3.3. Evaluation of Seedling Growth

The aboveground growth was assessed by determining biomass (fresh and dry weight), height, stem diameter, leaf area, and chlorophyll content; the underground growth was assessed by determining root biomass (fresh and dry weight) and root activity.

Height and stem diameter were measured using a ruler and a caliper gauge, respectively. To determine the leaf area, all leaves of each seedling were collected and photographed, of which the photos were analyzed using ImageJ software. The first and second leaves were collected to measure chlorophyll content by spectrophotometry. The underground growth was evaluated by determining root fresh and dry weights, and root activity. The whole root was collected to determined root activity using the 2,3,5-triphenyl tetrazolium chloride (TTC) method.

To determine aboveground and underground biomass, the seedlings were rinsed with water and the fresh weight was recorded after blotting the surface water. The seedlings were then transferred to a drying oven for a kill treatment at 105 °C for 30 min, followed by drying at 65 °C until a constant weight was reached for the dry weight.

#### 2.3.4. Determination of Chlorophyll Content

Chlorophyll content was measured using the method of Xiong et al. [28], with modifications. Briefly, 0.1 g fresh and clean leaf samples were soaked in 95% ethanol for 24 h to extract the chlorophyll. The leaves were collected after the first extraction and re-extracted, repeating this process three times. The extracts were combined, and the optical density (OD) was measured at 649 mm and 665 nm using a UV-VIS spectrophotometer (UV-2401, Shimadzu Corporation, Kyoto, Japan). The chlorophyll a (Chl a) and chlorophyll b (Chl b) contents were calculated using the following formulas, and the total chlorophyll content was obtained by the sum of Chl a and Chl b:

$$Chl\;a\;content\;(mg/L)\;=\;(13.95\times {OD}_{665}-6.88\times {OD}_{649})\times \frac{V}{m\times 1000}$$

$$Chl\;b\;content\;(mg/L)\;=\;(24.96\times {OD}_{649}-7.32\times {OD}_{665})\times \frac{V}{m\times 1000}$$

where *V* and *m* represent leaf sample weight and extract volume, respectively.

#### 2.3.5. Determination of Root Activity

The root activity was determined using the method described by Wang et al. [29]. Briefly, 0.5 g of roots was soaked in 5 mL of 0.4% TTC and 5 mL of 0.06 mol/L phosphate buffer (pH 7.0). After 3 h of reaction at 37 °C, 2 mL of 1 mol/L sulfuric acid was added to stop the reaction, followed by the addition of a color rendering agent, triphenyl formazan, to stain the roots. The roots were then collected and ground with a pestle in a mortar containing 4 mL of ethyl acetate and a small amount of quartz sand. The homogenate was allowed to stand at room temperature for 0.5 h, after which the liquid phase was collected and mixed with ethyl acetate. The OD was recorded at 485 nm (UV-2401, Shimadzu Corporation, Kyoto, Japan).

### 2.4. Determination of Antioxidant Enzyme Activities and MDA Content

The first and second leaves of each seedling were collected to determine the levels of superoxide dismutase (SOD), peroxidase (POD), catalase (CAT), and malondialdehyde (MDA). Briefly, 0.5 g of leaf samples was mixed with 100 mmol/L phosphate buffer solution (PBS) and ground using a mortar and pestle. The homogenate was brought to a final volume of 10 mL and centrifuged at 12,000 rpm for 20 min. The supernatant was collected for analysis, and subsequent steps were performed according to the method of Wang et al. [30]. Specifically, SOD activity was measured by the nitrogen blue tetrazolium reduction method, with absorbance read at 560 nm, following the method of Dhindsa et al. [31]. POD and CAT activities were measured by guaiacol method, with OD at 470 nm and 240 nm, respectively, according to the method of Rao et al. [32]. MDA content was measured by thiobarbituric acid method, with OD at 450 nm, 532 nm, and 600 nm, referring to the method of Dhindsa et al. [33].

### 2.5. Determination of Nutrient Conversion Enzyme Activities in Soil

The inter-root substrate was collected to measure urease, phosphatase, and sucrase activities using the method of by Guan et al. [34]. Sucrase activity was determined by the 3,5-dinitrosalicylic acid (DNS) colorimetric method. Briefly, 5 g of soil sample was mixed with 15 mL of 8% sucrose solution, 5 mL of PBS (pH 5.5), and 100 μL of toluene, and incubated at 37 °C for 24 h. After filtration, the filtrate was mixed with 3 mL of DNS and heated in a boiling water bath for 5 min. After cooling, the OD was measured at 510 nm.

Urease activity was determined by the sodium phenol-sodium hypochlorite colorimetric method. Briefly, 5 g of soil sample was mixed with 1.0 mL of toluene. After shaking for 15 min, 10 mL of 10% urea and 20 mL of citrate buffer (pH 6.7) were added, and the mixture was incubated at 37 °C for 24 h. The mixture was then filtered, and 1 mL of the filtrate was combined with 4 mL of sodium phenol and 3 mL of sodium hypochlorite. After a 20-min reaction, the OD was measured at 578 nm.

Phosphatase activity was determined by the disodium phosphate colorimetric method. Briefly, 5 g of soil sample was mixed with 2.5 mL of toluene, and 20 mL of 0.5% disodium phosphate solution was added after 15 min. After mixing, the sample was incubated at 37 °C for 24 h. Subsequently, 100 mL of 0.3% aluminum sulfate was added for 30 min of reaction. After filtration, the OD of the filtrate was measured at 660 nm.

### 2.6. Statistical Analysis

In the antimicrobial experiment, three Petri dishes were used as replicates. In the potting experiment, each group consisted of three blocks as replicates with 30 pots per block. To evaluate the seedling growth, five seedlings were randomly selected in each block and the average value was used. Data were analyzed using Duncan’s multiple range test [35] and Student’s *t*-test [36] in SPSS v22.0 software (IBM Institute, Armonk, NY, USA). A *p*-value of <0.05 was considered statistically significant.

## 3. Results and Discussion

### 3.1. Effect of SGFP on Inhibition of F. oxysporum Causing Plant Wilt Disease

**Figure 1:**
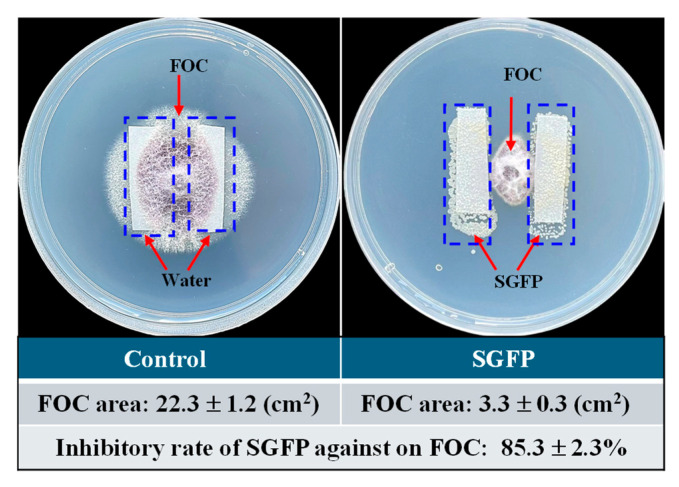
Effect of *Streptomyces rochei* G-6 fermentation product (SGFP) on antimicrobial activity against *Fusarium oxysporum* f. sp. *cucumerinum* (FOC). Data represent the mean ± standard deviation (*n* = 3).

*Streptomyces* species are known to produce antibiotics and antimicrobial compounds, as demonstrated in many studies [37,38,39]. In this study, the growth of FOC was inhibited during co-incubation with SGFP. As shown in Figure 1, the control group exhibited 22.3 ± 1.2 m^2^ coverage of FOC, while the SGFP-treated group showed minimal growth at 3.3 ± 0.3 cm^2^, resulting in an inhibition rate of 85.3 ± 2.3%.

The results of this study were consistent with previous findings that *Streptomyces* metabolites can effectively inhibit *F. oxysporum*, which causes wilt disease in different plants. For instance, Le et al. [40] demonstrated that the fermentation broth of *Streptomyces* sp. AN090126 significantly suppresses the growth of *F. oxysporum* f. sp *lycopersici*, a pathogen responsible for tomato wilt disease, due to the presence of antifungal compounds in the fermentation broth, such as 3-methyl-1-butanol, 2-propyl furan, and dimethyl disulfide. Qi et al. [41] observed significant antifungal activity of *Streptomyces* sp. SCA3-4 against *F. oxysporum* f. sp. *cubense*, which causes banana wilt disease. The observed inhibition rate of 85.3% against FOC in this study suggests that SGFP may contain potent antifungal metabolites that could serve as effective natural biocontrol agents against cucumber wilt disease. This observation provides a basis for the subsequent biocontrol experiment.

### 3.2. Effect of SGFP on Preventing and Treating Cucumber Wilt Caused by FOC

The biocontrol effect of SGFP against cucumber wilt disease was evaluated from both preventive (SGFP1) and treatment (SGFP2) perspectives. As shown in Table 1, no disease symptoms were observed in the control group after 14 days of cultivation, confirming that the experimental conditions were pathogen-free.

**Figure 2:**
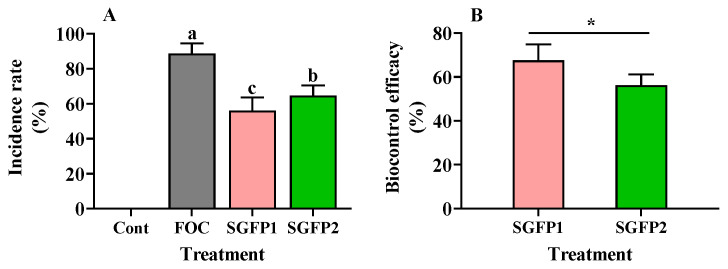
Effect of *Streptomyces rochei* G-6 fermentation product (SGFP) on wilt incidence rate of wilt (**A**) and biocontrol efficacy (**B**) after 14 days of cucumber seedling cultivation in pots. Cont = Control group. FOC = *Fusarium oxysporum* f. sp. *cucumerinum* disease group. SGFP1 = Preventive group treated with SGFP. SGFP2 = Treatment group treated with SGFP. Data represent the mean ± standard deviation (*n* = 3). Different letters on bars indicate significant differences at *p* < 0.05 according to Duncan’s multiple test. An asterisk (*) indicates a significant difference at *p* < 0.05 by Student’s *t*-test.

In the FOC group, a high disease index of 64.8 was recorded, with an average of 10.6 out of 30 seedlings reaching Grade 4, indicating severe infection. In contrast, the SGFP1 group reduced the disease index to 21.0, with an average of 13.2 uninfected seedlings and no cases of Grade 4 infection. No cases of Grade 4 infection were also observed in the SGFP2 group. The disease index in SGFP2 reached 28.3, which was higher than that in SGFP1 group but lower than that in FOC group. In addition, an average of 10.6 seedlings remained uninfected in SGFP2; however, the number of seedlings with Grade 3 infection increased compared to the SGFP1 group, suggesting that SGFP is more effective as a prevention strategy than as a treatment strategy. The wilt incidence rate in the FOC group reached 88.7%, which decreased to 56.0% and 64.7% in SGFP1 and SGFP2 groups, respectively (Figure 2A). The biocontrol efficacy was 67.6% for SGFP1 and 56.3% for SGFP2, further illustrating the greater effectiveness of the preventive strategy (Figure 2B).

*Streptomyces* sp. have been demonstrated to effectively resist wilt disease in various studies. For example, Wang et al. [42] treated pot-cultivated banana seedlings with a fermentation broth of *Streptomyces* sp. XY006 and found a high control efficacy of 87.7%. Kawicha et al. [43] also found that the fermentation filtration of *Streptomyces* sp. STRM103 decreased disease index of tomato wilt, with a control efficacy of 60.7%. In the case of cucumber wilt disease, Le et al. [40] revealed that the fermentation broth of *Streptomyces* sp. JCK-6131 inhibited wilt in a dose-dependent manner, achieving a high control efficacy of 83.3% in the high-dose (five-fold) group, which was comparable to that in the positive control group. These findings suggest the potential of *Streptomyces* fermentation products against wilt diseases in different plants and indicate that the control efficiency depends on the *Streptomyces* strains, attributable to their varying metabolites.

**Figure 3:**
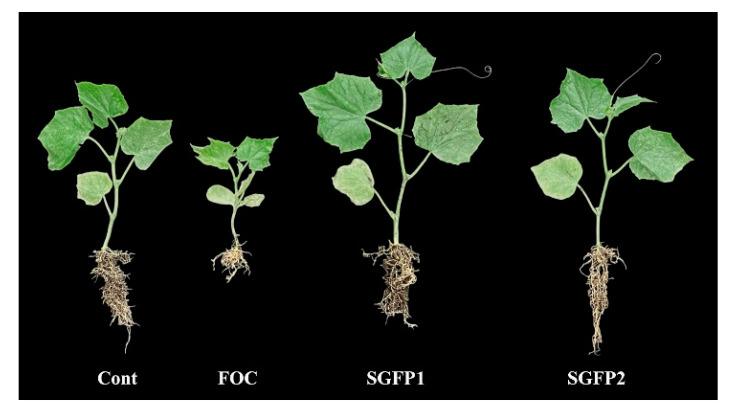
Cucumber seedling growth in different groups after 14 days of cultivation in pots. Cont = Control group. FOC = *Fusarium oxysporum* f. sp. *cucumerinum* disease group. SGFP1 = Preventive group treated with SGFP. SGFP2 = Treatment group treated with SGFP.

In this study, *S. rochei* was used for the first time to control cucumber wilt. Yellowing leaves were observed in some FOC-infected seedlings after seven days, followed by wilted apical buds after 14 days. However, these symptoms were alleviated by SGFP treatment. SGFP1 showed superior biocontrol efficacy in reducing cucumber wilt, especially at higher infection levels (Grade 3 and Grade 4). Although SGFP2 was effective, early application (SGFP1) proved to be the more beneficial strategy (Figure 3). Further studies should optimize the SGFP concentration and timing, and compare the effect with the chemical agents (as a positive control) commonly used to determine the optimal SGFP application strategy.

### 3.3. Effect of SGFP on Enhancement of Cucumber Seedling Growth

In addition to its strong biocontrol effects against cucumber wilt, SGFP also promoted the growth of cucumber seedlings. As shown in Table 2, aboveground biomass (fresh and dry weight) in the FOC group was significantly reduced (*p* < 0.05) compared to the control, while both SGFP1 and SGFP2 groups exhibited significantly (*p* < 0.05) higher biomass than both the FOC and control groups. Seedling height was reduced in the FOC group, indicating disease inhibition, whereas SGFP1 and SGFP2 resulted in significant (*p* < 0.05) height increases, with SGFP1 showing the strongest effect. Stem diameter did not differ significantly (*p* > 0.05) between the control and FOC groups, but the SGFP1 and SGFP2 groups had greater stem thickness, especially SGFP1. Similarly, the leaf area was smaller in the FOC group than in the control, but SGFP1 and SGFP2 resulted in significantly (*p* < 0.05) larger leaf areas, indicating that SGFP promotes leaf expansion and potentially enhances photosynthesis. Chlorophyll content followed a similar trend, with SGFP1 and SGFP2 showing higher levels (*p* < 0.05), indicating improved photosynthetic efficiency.

SGFP also positively affected the root growth. Both root fresh and dry weights were significantly lower (*p* < 0.05) in the FOC group, but SGFP1 and SGFP2 restored root biomass to control levels, showing a significant improvement over FOC (*p* < 0.05). Root activity was similarly enhanced, with SGFP1 showing the strongest effect (Table 3).

Similar to the results of our study, several investigations have reported the growth-enhancing effects of *Streptomyces* on plants, often accompanied by biocontrol effects. For instance, the fermentation broth of *S. rimosus* M527 and the fermentation supernatant of *Streptomyces* sp. CNS-42 has been shown to significantly increase cucumber shoot biomass and height when used as biocontrol agents against cucumber wilt disease [44,45]. These findings demonstrate that *Streptomyces* not only provides biocontrol against cucumber wilt but also promotes plant growth. This enhancement is likely attributed to the presence of growth-promoting compounds, such as auxins and cytokinins, found in *Streptomyces* metabolites. Additionally, the increased growth of cucumber seedlings observed in this study may activate mechanisms such as antioxidant system regulation and enhanced nutrient conversion in the soil.

### 3.4. Effect of SGFP on Antioxidant System of Cucumber Seedlings

**Figure 4:**
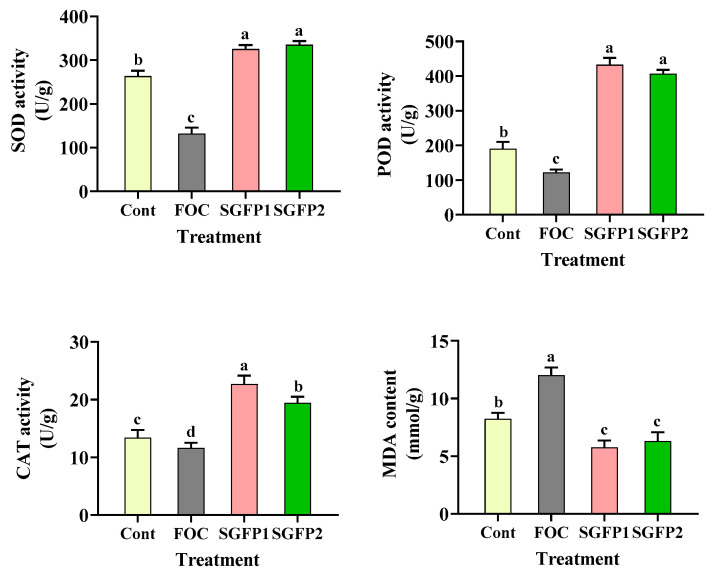
Effect of *Streptomyces rochei* G-6 fermentation product (SGFP) on antioxidant enzyme and malondialdehyde levels after 14 days of cucumber seedling cultivation in pots. Cont = Control group. FOC = *Fusarium oxysporum* f. sp. *cucumerinum* disease group. SGFP1 = Preventive group treated with SGFP. SGFP2 = Treatment group treated with SGFP. SOD = Superoxide dismutase. POD = Peroxidase. CAT = Catalase. MDA = Malondialdehyde. Data represent the mean ± standard deviation (*n* = 3). Different letters in the column indicate significant differences at *p* < 0.05 according to Duncan’s multiple test.

To investigate whether SGFP promotes cucumber seedling growth by regulating the antioxidant system, we measured the activities of SOD, POD, CAT, and MDA content in the seedling leaves. Figure 4 shows that the activities of the three antioxidant enzymes were significantly higher (*p* < 0.05) in the SGFP group than in the control group, indicating that SGFP infection impaired the antioxidant defense of cucumber seedlings. The sharp decrease in SOD, POD, and CAT activities in the FOC group reflected the severe oxidative stress caused by the disease. In contrast, significantly higher (*p* < 0.05) antioxidant enzyme activities were found in the SGFP1 and SGFP2 groups compared to the FOC and control groups, indicating that SGFP treatment promotes the antioxidant capacity of cucumber seedlings. In addition, MDA levels were highest in the FOC group, indicating membrane lipid peroxidation due to oxidative stress, consistent with the result of Du et al. [46] linking high MDA levels to membrane damage and increased oxidative injury.

This observation supports the findings of Li et al. [47] that biocontrol agents increase antioxidant enzyme activity under stress, thereby improving plant resistance. Increased enzyme activity can help detoxify reactive oxygen species and protect cellular structures from damage.

Conversely, the significant reduction (*p* < 0.05) in MDA in the SGFP1 and SGFP2 groups illustrates the protective role of SGFP in minimizing membrane damage and oxidative stress. This reinforces the importance of antioxidant defenses in maintaining cellular integrity during pathogen attack, as shown by Jones et al. [48].

This study suggests that SGFP can alleviate oxidative stress by increasing antioxidant enzyme activities and reducing MDA accumulation, thereby contributing to seedling growth. Future research should focus on the underlying mechanisms of SGFP in regulating antioxidant pathways and its synergistic effects with other biocontrol agents to further improve plant health and disease resistance.

### 3.5. Effect of SGFP on Nutrient Conversion in Soli

**Figure 5:**
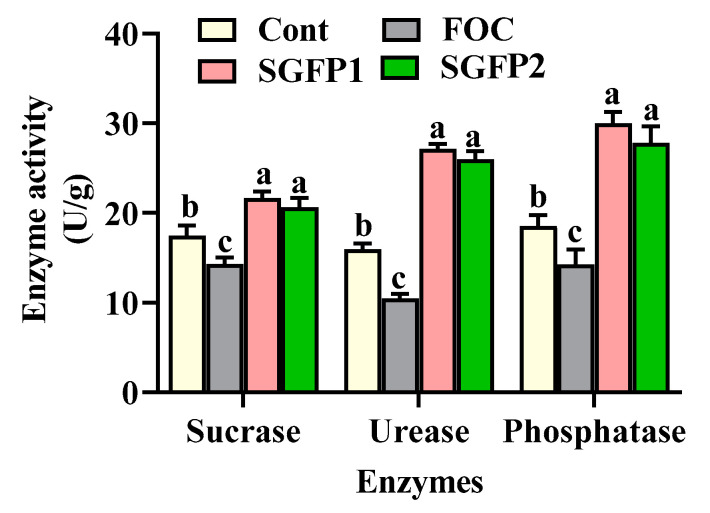
Effect of *Streptomyces rochei* G-6 fermentation product (SGFP) on activities of soil nutrient-conversion enzymes after 14 days of cucumber seedling cultivation in pots. Cont = Control group. FOC = *Fusarium oxysporum* f. sp. *cucumerinum* disease group. SGFP1 = Preventive group treated with SGFP. SGFP2 = Treatment group treated with SGFP. Data represent the mean ± standard deviation (*n* = 3). Different letters in each enzyme indicate significant differences at *p* < 0.05 according to Duncan’s multiple test.

This study also examined the effect of SGFP on nutrient conversion enzyme activities, including sucrase (carbohydrate degradation), urease (nitrogen conversion), and phosphatase (phosphorus conversion) in the cucumber pot substrate. As shown in Figure 5, three enzyme activities in the FOC group were significantly lower than in the control group (*p* < 0.05), indicating that FOC negatively affected soil enzyme activities. In contrast, enzyme activities in the SGFP1 and SGFP2 groups were significantly higher (*p* < 0.05) than both the FOC and control groups, suggesting that SGFP effectively restores or enhances nutrient-conversion processes.

The findings are consistent with Paolo et al. [49], who found that biocontrol agents improve soil enzyme activities, thereby increasing nutrient availability to plants. The similar enzyme activity levels between SGFP1 and SGFP2 indicate that SGFP is effective in both preventive and treatment applications. Furthermore, the increased sucrase activity in this study suggests improved carbohydrate breakdown, supporting microbial activity and nutrient cycling, while enhanced urease and phosphatase activities indicate improved nitrogen and phosphorus availability, essential for plant growth. These results demonstrate that SGFP plays a dual role in controlling cucumber wilt and promoting seedling growth by boosting enzyme activity and nutrient transformation.

**Figure 6:**
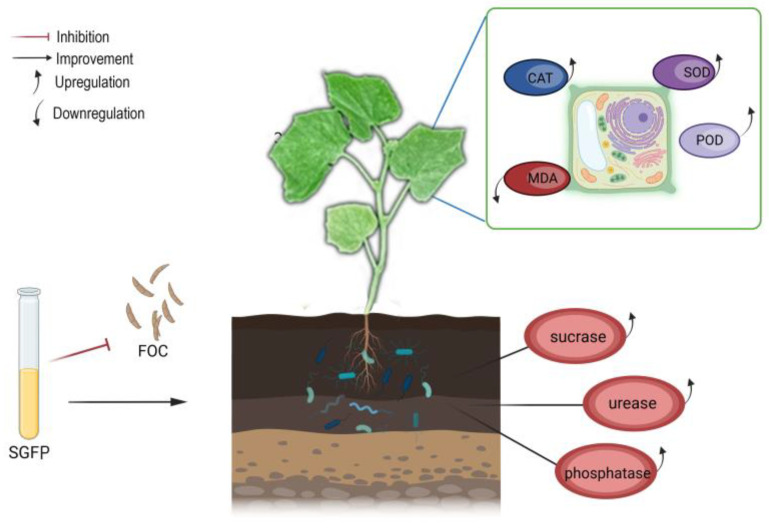
Passible mechanisms of *Streptomyces rochei* G-6 fermentation product (SGFP) in controlling wilt disease and promoting growth of cucumber.

From the results of this study, we concluded the possible mechanisms of SGFP on controlling cucumber wilt disease and promoting growth as: *S. rochei* can produce antimicrobials and plant growth-promoting compounds [17,18]. The presence of antimicrobials in SGFP inhibits FOC and exerts the biocontrol effect on cucumber wilt disease. In addition, SGFP can activate the antioxidant system of cucumber seedlings under FOC stress, thereby increasing the activities of SOD, POD, and CAT while reducing the MDA content to avoid the negative effect of FOC. On the other hand, SGFP may improve the microbial community in the root zone, thereby increasing the activities of nutrient conversion enzymes such as sucrase, urease, and phosphatase, thereby increasing the nutrient utilization rate, and consequently promoting underground growth, which ultimately enhances aboveground growth (Figure 6).

This study indicates the potential of SGFP in agriculture; however, several questions remain to be addressed, including the optimal application method and concentration of SGFP in different soil types and cropping systems, which is the next step in our strategy. In addition, we believe that future efforts should focus on further screening and characterizing the metabolite compounds produced by *S. rochei* G-6, investigating its growth-promoting mechanisms, optimizing fermentation conditions, conducting field trials to validate the efficacy, and assessing the long-term effects and safety. These steps will not only help confirm the potential of *S. rochei* G-6 in agricultural applications, but also support its commercialization as a biocontrol agent and plant growth promoter.

## 4. Conclusions

SGFP inhibits the growth of FOC, with an inhibition rate of 85.3%. Furthermore, both preventive (SGFP1) and treatment (SGFP2) applications of SGFP reduce disease index and incidence rates, with SGFP1 of the most effective strategy. In addition, SGFP treatment improves seedling growth with increased antioxidant enzyme activities and reduces MDA levels, indicating its protective role against oxidative stress in cucumber seedlings. In addition, SGFP positively affects soil nutrient conversion processes by increasing the activities of urease, phosphatase, and sucrase, thereby promoting nutrient availability and further growth of seedlings.

The findings of this study suggest that SGFP is a promising biocontrol agent for the control of cucumber wilt disease while improving soil health and nutrient cycling. Future studies should focus on optimizing SGFP application protocols to maximize its effectiveness in different agricultural settings.

## Figures and Tables

**Table 1:** Effect of *Streptomyces rochei* G-6 fermentation product (SGFP) on wilt disease index after 14 days of cucumber seedling cultivation in pots.

Treatment	Number of Seedlings					Disease Index
	Grade 0	Grade 1	Grade 2	Grade 3	Grade 4	
Cont	30.0 ± 0.0 a	0.0 ± 0.0 c	0.0 ± 0.0 d	0.0 ± 0.0 d	0.0 ± 0.0 b	0.0 ± 0.0 d
FOC	3.4 ± 0.6 d	4.0± 1.0 b	4.6 ± 1.5 b	7.4 ± 1.5 a	10.6 ± 1.5 a	64.8 ± 6.0 a
SGFP1	13.2 ± 0.6 b	10.6 ± 0.5 a	4.0 ± 0.0 c	2.2 ± 0.4 c	0.0 ± 0.0 b	21.0 ± 1.3 c
SGFP2	10.6 ± 0.5 c	9.4 ± 0.6 a	5.4 ± 0.6 a	4.6 ± 0.6 b	0.0 ± 0.0 b	28.3 ± 2.2 b

Cont = Control group. FOC = *Fusarium oxysporum* f. sp. *cucumerinum* disease group. SGFP1 = Preventive group treated with SGFP. SGFP2 = Treatment group treated with SGFP. Data represent the mean ± standard deviation (*n* = 3). Different letters in the column indicate significant differences at *p* < 0.05 according to Duncan’s multiple test.

**Table 2:** Effect of *Streptomyces rochei* G-6 fermentation product (SGFP) on aboveground growth after 14 days of cucumber seedling cultivation in pots.

Treatment	Biomass (g/Seedling)		Height (cm)	Stem Diameter (mm)	Leaf Area (cm^2^)	Chlorophyll Content (mg/g)
	Fresh Weight	Dry Weight				
Cont	3.9 ± 0.7 b	0.36 ± 0.1 c	19.1 ± 0.7 b	4.2 ± 0.2 b	54.6 ± 2.9 c	0.9 ± 0.1 b
FOC	2.4 ± 0.6 c	0.2 ± 0.1 d	8.9 ± 1.3 c	3.8 ± 0.4 b	34.8 ± 3.9 d	0.8 ± 0.1 b
SGFP1	5.2 ± 0.3 a	0.6 ± 0.1 a	28.6 ± 2.9 a	4.9 ± 0.3 a	78.3 ± 4.3 a	1.3 ± 0.3 a
SGFP2	4.9 ± 0.4 a	0.5 ± 0.1 b	25.1 ± 2.1 a	4.3 ± 0.2 ab	62.4 ± 1.8 b	1.2 ± 0.2 a

Cont = Control group. FOC = *Fusarium oxysporum* f. sp. *cucumerinum* disease group. SGFP1 = Preventive group treated with SGFP. SGFP2 = Treatment group treated with SGFP. Data represent the mean ± standard deviation (*n* = 3). Different letters in the column indicate significant differences at *p* < 0.05 according to Duncan’s multiple test.

**Table 3:** Effect of *Streptomyces rochei* G-6 fermentation product (SGFP) on underground growth after 14 days of cucumber seedling cultivation in pots.

Treatment	Biomass (g/Seedling)		Root Activity mg/(g·h)
	Fresh Weight	Dry Weight	
Cont	0.6 ± 0.1 a	136.7 ± 10.6 b	0.4 ± 0.1 c
FOC	0.3 ± 0.1 b	67.9 ± 5.7 c	0.2 ± 0.1 d
SGFP1	0.7 ± 0.3 a	157.2 ± 9.7 a	0.7 ± 0.1 a
SGFP2	0.6 ± 0.2 a	120.6 ± 8.6 b	0.6 ± 0.1 b

Cont = Control group. FOC = *Fusarium oxysporum* f. sp. *cucumerinum* disease group. SGFP1 = Preventive group treated with SGFP. SGFP2 = Treatment group treated with SGFP. Data represent the mean ± standard deviation (*n* = 3). Different letters in the column indicate significant differences at *p* < 0.05 according to Duncan’s multiple test.

## Data Availability

The original contributions presented in this study are included in the article. Further inquiries can be directed to the corresponding authors.
